# Posterior Cruciate Ligament Retention versus Posterior Stabilization for Total Knee Arthroplasty: A Meta-Analysis

**DOI:** 10.1371/journal.pone.0147865

**Published:** 2016-01-29

**Authors:** Chao Jiang, Zhenlei Liu, Ying Wang, Yanyan Bian, Bin Feng, Xisheng Weng

**Affiliations:** 1 Department of Orthopedic Surgery, Peking Union Medical College Hospital, Chinese Academy of Medical Sciences and Peking Union Medical College, Beijing, 100730, China; 2 Department of Ultrasound, Peking Union Medical College Hospital, Chinese Academy of Medical Sciences and Peking Union Medical College, Beijing, 100730, China; Mayo Clinic Minnesota, UNITED STATES

## Abstract

**Introduction:**

Although being debated for many years, the superiority of posterior cruciate-retaining (CR) total knee arthroplasty (TKA) and posterior-stabilized (PS) TKA remains controversial. We compare the knee scores, post-operative knee range of motion (ROM), radiological outcomes about knee kinematic and complications between CR TKA and PS TKA.

**Methods:**

Literature published up to August 2015 was searched in PubMed, Embase and Cochrane databases, and meta-analysis was performed using the software, Review Manager version 5.3.

**Results:**

Totally 14 random control trials (RCTs) on this topic were included for the analysis, which showed that PS and CR TKA had no significant difference in Knee Society knee Score (KSS), pain score (KSPS), Hospital for Special Surgery score (HSS), kinematic characteristics including postoperative component alignment, tibial posterior slope and joint line, and complication rate. However, PS TKA is superior to CR TKA regarding post-operative knee range of motion (ROM) [Random Effect model (RE), Mean Difference (MD) = -7.07, 95% Confidential Interval (CI) -10.50 to -3.65, *p*<0.0001], improvement of ROM (Fixed Effect model (FE), MD = -5.66, 95% CI -10.79 to -0.53, *p* = 0.03) and femoral-tibial angle [FE, MD = 0.85, 95% CI 0.46 to 1.25, *p*<0.0001].

**Conclusions:**

There are no clinically relevant differences between CR and PS TKA in terms of clinical, functional, radiological outcome, and complications, while PS TKA is superior to CR TKA in respects of ROM, while whether this superiority matters or not in clinical practice still needs further investigation and longer follow-up.

## Introduction

Although being debated for many years, the superiority of posterior cruciate-retaining (CR) total knee arthroplasty (TKA) and posterior-stabilized (PS) TKA remains controversial. With the posterior cruciate ligament (PCL) retained, CR TKA was thought to be better regarding post-operative knee proprioception and kinesthesia [1,2]. While others believed that PS TKA had better range of motion (ROM) [3], easier in ligament balance, and more reliable femoral rollback [4,5]. A systematic review and meta-analysis from 2012 compared knee scores, ROM, radiographic kinematics and complication between these two TKA designs, indicating that CR and PS TKA had no differences in knee scores, radiological outcomes and complications. Although PS TKA had a better ROM, it made no clinical difference [6]. Following updates were published in 2013 [7] and 2014 [8] reporting similar outcomes in knee score and function. These studies included related random control trials (RCTs) till August 2011. Since then, there were more RCTs published to compare CR and PS TKA in clinical knee scores, function and complications [9–13]. Taking this into consideration, we think it is necessary to make an update on this topic. The outcome measures for data aggregation were knee scores, post-operative ROM, radiological outcomes about knee kinematics and complications.

## Methods

### 2.1 Eligibility criteria

(1) RCTs with at least 6 months follow-up. (2) Participants underwent primary TKA, unilateral or bilateral. (3) The operations were performed with PCL Retaining versus Posterior Stabilized prosthesis. The studies that compare PCL retaining versus sacrificing TKA using the same prosthesis were excluded. (4) End points were about clinical knee scores, clinical function, kinematic characteristics and complications.

### 2.2 Literature search

Literature published up to August 2015 was searched in PubMed, Embase and Cochrane databases. We used key words “Total Knee Arthroplasty”, “Posterior Cruciate Ligament Retention”, “Posterior Cruciate Ligament Retaining”, “Posterior Stabilization”, “Posterior Cruciate Ligament Sacrificing”, and their synonyms to retrieve all studies about this topic. We also reviewed references of related reviews so that no studies were missed.

### 2.3 Study selection

Title and abstract review was conducted firstly to rule out the apparently unrelated articles. Then the articles would be examined through the text to determine whether they should be included for the meta-analysis or not according to the eligibility criteria. Reviews and former meta-analyses about this topic were also kept for reference review. All screening works were conducted independently by two authors. Disagreements were discussed and consulted with corresponding author until a consensus was made.

### 2.4 Data extraction

Each study included was reviewed thoroughly to extract as much data as we can. Clinical scores, including Knee Society knee Score (KSS), function score (KSFS) and pain score (KSPS), The Western Ontario and McMaster Universities score (WOMAC) and Hospital for Special Surgery score (HSS), clinical function, including ROM, postoperative knee extension and flexion, kinematic characteristics, including postoperative component alignment, tibial posterior slope and joint line, and complications were all in the scope of this meta-analysis. With incomplete data in the published articles (e.g. only mean and range for specific measurements), we attempted to contact the authors for original data so that we can include more patients in this analysis.

### 2.5 Statistical analysis

We use the software, Review Manager (RevMan) version 5.3, which is for Cochrane reviews, to perform this meta-analysis. With RevMan, publication bias was visually inspected with the funnel plot, quality assessment was conducted with the risk and bias tables, and heterogeneity of included studies was tested with Chi^2^ and heterogeneity index, I^2^.

For clinical scores, function and kinematic characteristics, which are continuous, we employed FE model and the Inverse Variance method. For complications, which are dichotomous, we employed the FE model and the Mantel-Haenszel method. We defined that any complication that need to remove the prosthesis or re-surgery as a severe complication, and others as mild ones. RE model was used if subgroup and sensitivity analyses cannot settle heterogeneity issue. For each measurement, 95% CI and *p* value were calculated. *p* <0.05 was considered statistically significant.

## Results

### 3.1 Characteristics of included studies

**Fig 1:**
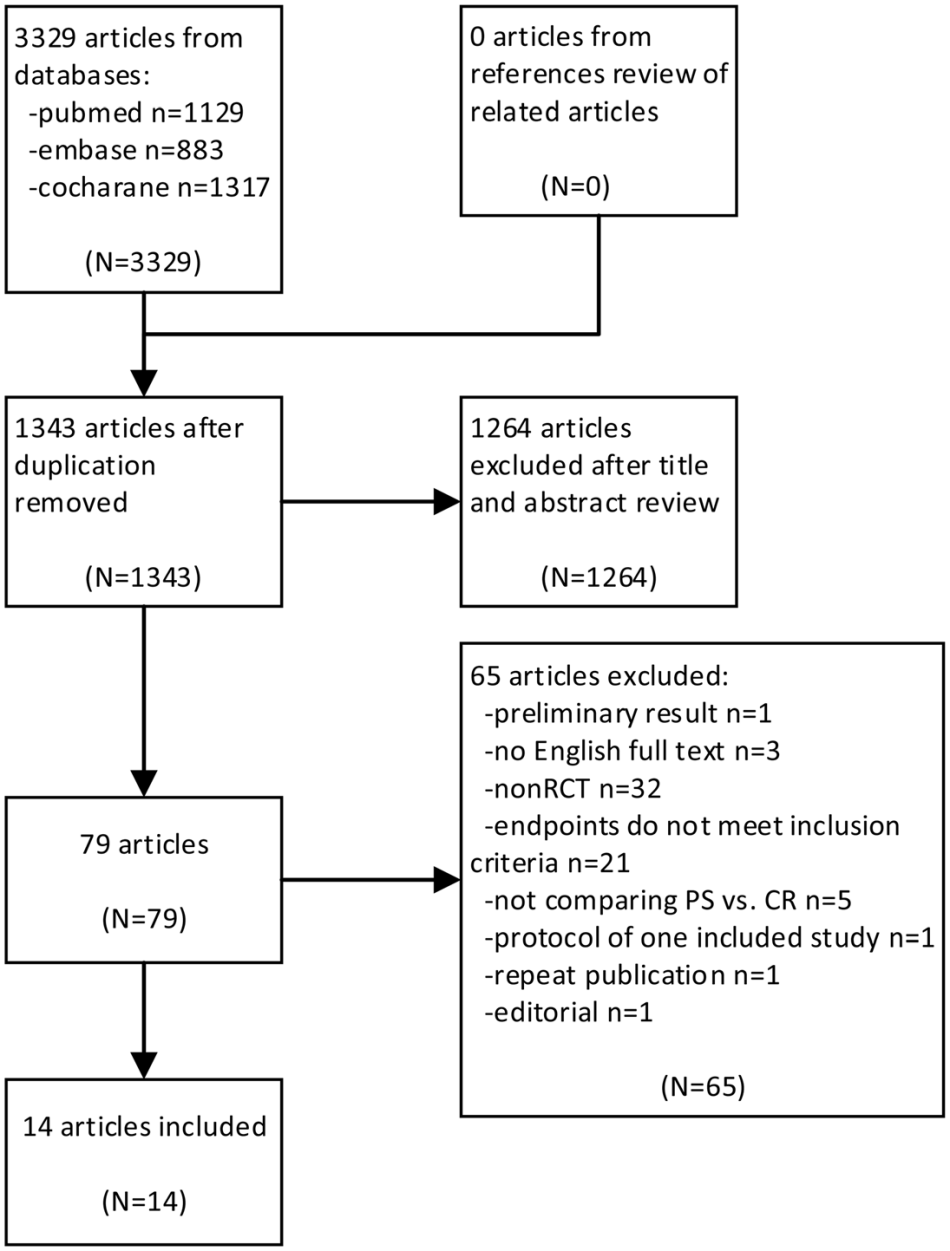
Flow diagram of study selection.

The details of literature search strategies and the corresponding results are available in S1 File. Totally 3329 articles were retrieved from the three databases. The screening process was shown in Fig 1. Finally we included 14 studies with 791 patients underwent TKA with CR prosthesis and 662 patients with PS prosthesis. The basic characteristics of these studies were summarized in Table 1.

**Table 1:** Basic characteristics of included studies.

No.	Sample size			TKA		Mean age(y)		Male(%)		Mean BMI (or weight)		Brand of prostheses	Outcomes	Complications		Follow-up
	patients	knees	OA %	CR	PS	CR	PS	CR	PS	CR	PS			CR	PS	
Clark [14] 2001	128	128	97	59	59	71.8±12.2	71.2±13.6	ND	ND	83.3 ±31.6	82.1±38.4	Johnson & Johnson	KSS, WOMAC, SF-12, ROM	ND	ND	>1 year
Tanzer [15] 2002	37	40	97	20	20	68	66	25	20	185	174	Zimmer	KSS, KSFS, Flexion, Radiographs, Complications	5 non-progressive radiolucencies	8 non-progressive radiolucencies	2 years
Catani [16] 2004	40	40	100	20	20	70±6	71±7	35	25	ND	ND	Exactech	HSS, KSS, Radiographs, Complications	1 lateral release and patella resurfacing for anterior knee pain, 1 manipulation for limited ROM	2 lateral release and patella resurfacing for anterior knee pain	2 years
Maruyama [17] 2004	20	40	100	20	20	74	74	40	40	ND	ND	Johnson & Johnson	KSS, Radiographs, Complication	0	1 superficial wound infection	>2 years
Wang [18] 2004	228	267	91	157	110	54.5	55	19.7	19.8	65.9±10.7	63.7±11.0	Johnson & Johnson	KSS, KSFS, SF-12, ROM, Flexion/Extension, Radiographs	ND	ND	42 months (24–66 months)
Chaudhary [19] 2008	100	100	ND	51	49	69.2±9.1	70.2±8.4	47	55	32.4±5.7	30.9±4.3	Stryker	Flexion/Extension angle, KSFS, Pain score, Complications	1 removal of the implants for infection	1 manipulation for poor knee flexion	22.7±5.2months
Harato [3] 2008	189	192	100	99	93	68.3	66	34.3	34.3	29.8	31.4	Smith & Nephew	KSS, KSPS, WOMAC, ROM, SF-12, Complications	1 lucent line, 1 infection underwent revision, 7 stiff knee, 2 hemoarthrosis, 5 anterior knee pain, 1 0ther	1 lucent line, 1 DVT, 3 infection underwent revision, 1 stiff knee, 1 hemoarthrosis, 2 anterior knee pain, 4 others	>5 years
Kim [20] 2009	250	500	100	250	250	71.6±6	71.6±6	4	4	26.8±3.2	26.8±3.2	Zimmer	KSS, HSS, WOMAC, Flexion, Radiographs, Complications	2 anterior femoral notching, 1 superficial wound infection	3 anterior femoral notching, 1 superficial wound infection	2.3 years
Seon [21] 2011	95	95	100	48	47	68.2±7	69.2±6.7	8.3	10.6	25.8±3.4	23.7±2.8	Zimmer	HSS, WOMAC, ROM, Radiographs	ND	ND	27 months
Matsumoto [9] 2012	41	41	100	19	22	73.5±1.3	74.4±0.9	0	0	ND	ND	Zimmer	KSS, KSFS, ROM, Flexion, Extension, Radiographs	ND	ND	5 years
Yagishita [10] 2012	29	58	100	29	29	74.3±7.2	74.3±7.2	13.8	13.8	26.3±3	26.3±3	Zimmer	KSS, KSFS, KSPS, ROM, Flexion/Extension, Radiographs, Complications	1 DVT	0	5 years
Thomsen [11] 2013	36	72	97	36	36	67	67	58	58	29.4	29.4	Zimmer, Biomet-Merck	VAS, Flexion, SF-36, Complications	1 infection underwent revision	0	1 year
van den Boom [12] 2014	21	21	100	9	12	72±8	75±6	77.8	41.7	ND	ND	Warsaw	KSS, WOMAC, ROM, Flexion/Extension, Gait analysis, Knee kinematics	ND	ND	6–9 months
Vermesan [13] 2015	50	50	ND	50	50	68.8±6.9	68.4±6.3	60	88	32.6±7.1	33.4±7.5	Biomet, Zimmer	KSS, WOMAC, ROM, Complications	3 stiff knee	1 stiff knee, 1 infection treated with drainage and antibiotic	6 months

### 3.2 Quality assessment and publication bias inspection

**Fig 2:**
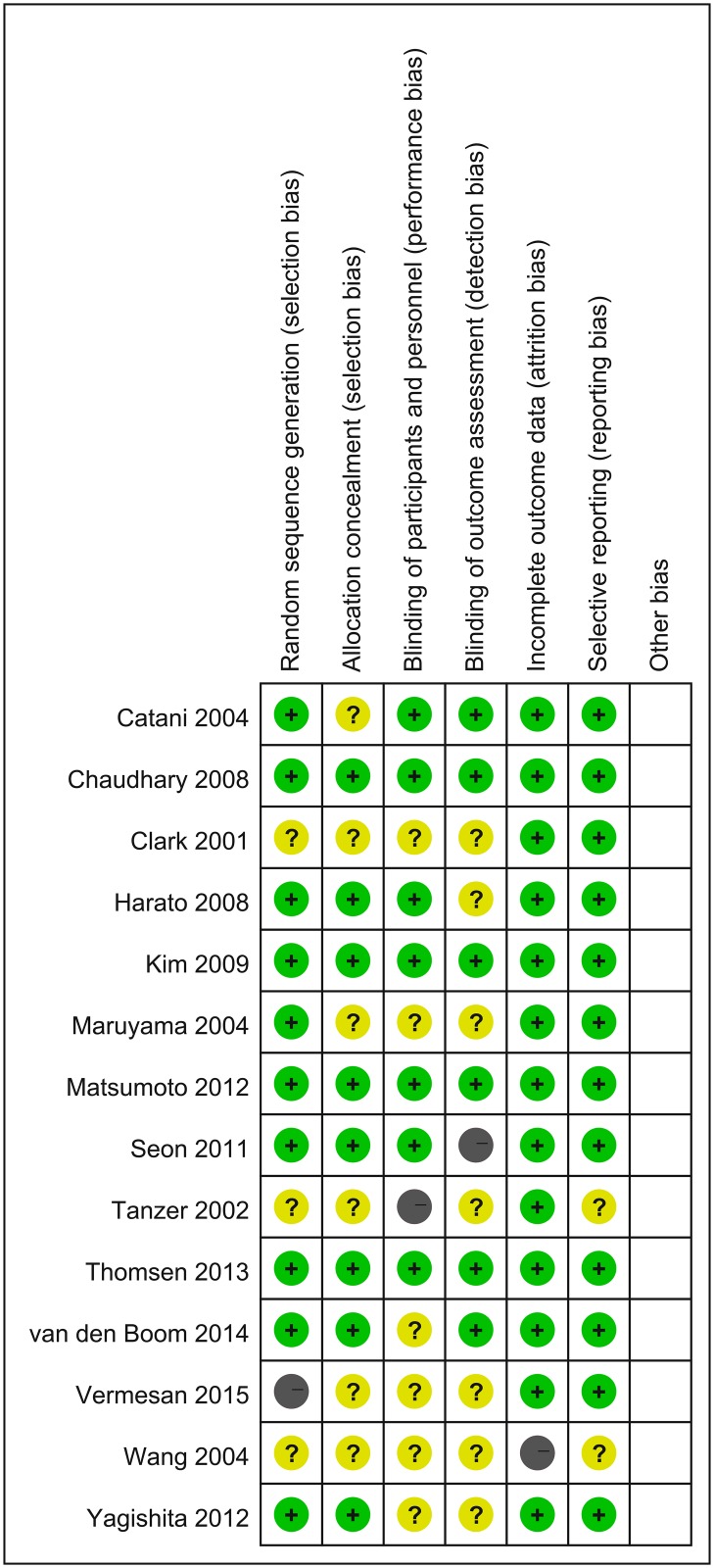
Quality assessment summary. Grey with a minus sign: High risk; Yellow with a question mark: Unclear risk; Green with a plus sign: Low risk. Graded according to the instruction in RevMan software.

**Fig 3:**
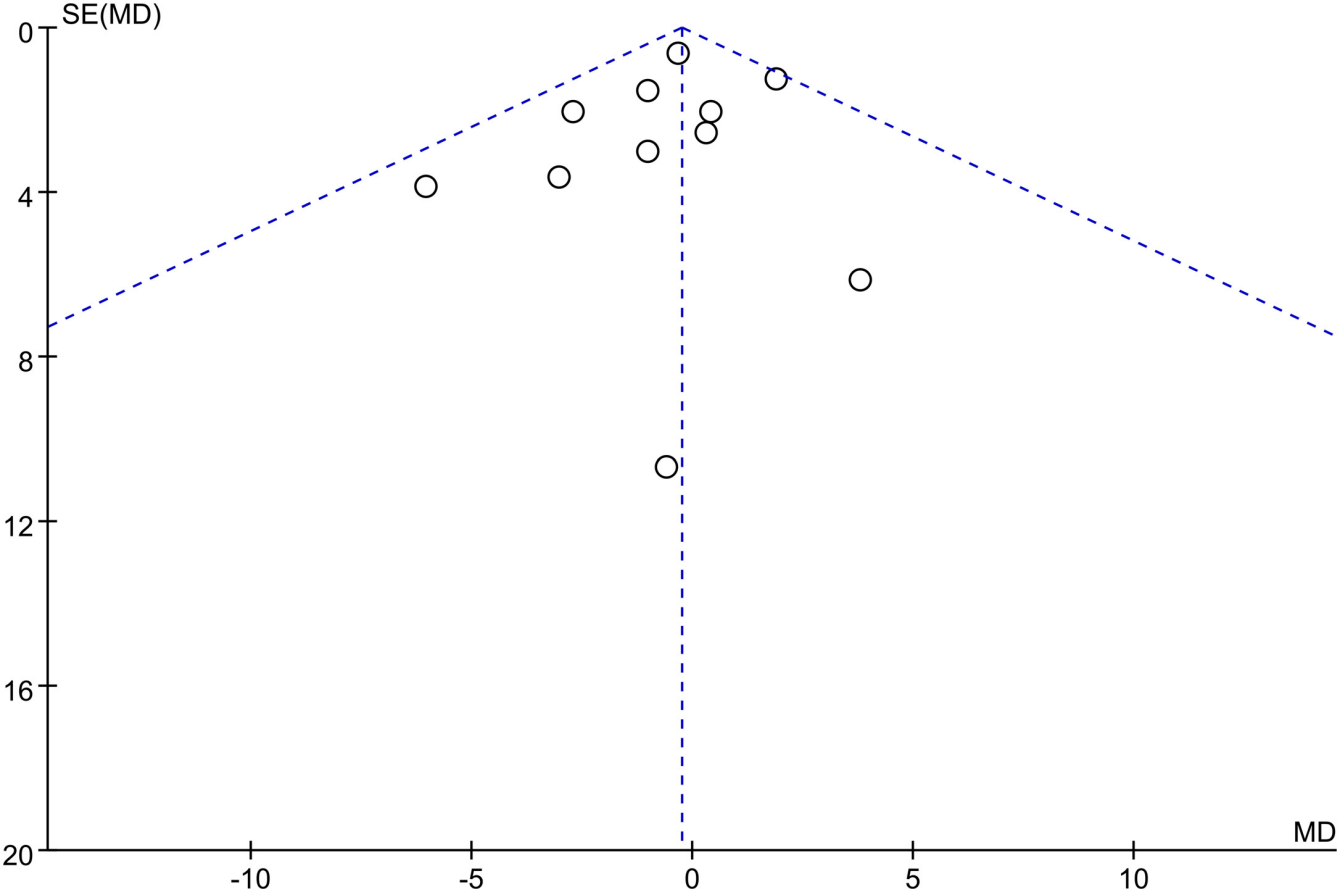
Funnel plot for publication bias inspection. All included studies are within the dotted line, indicating no significant publication bias among the studies.

The quality assessment were performed with the risk and bias table in RevMan and summarized in Fig 2, as we could see, most of the articles were low to moderate risk according to quality assessment. The reasons for each judgement are available in S2 File. We can conclude that most RCTs were performed with a relatively high quality. Publication bias was visually inspected with funnel plot in RevMan (Fig 3). We use the analysis of KSS to generate this funnel plot because it included 11 of 14 studies and covered more than any other analysis. Fig 3 showed there was no significant publication bias among these studies.

### 3.3 Meta-analysis of the clinical scores

**Fig 4:**
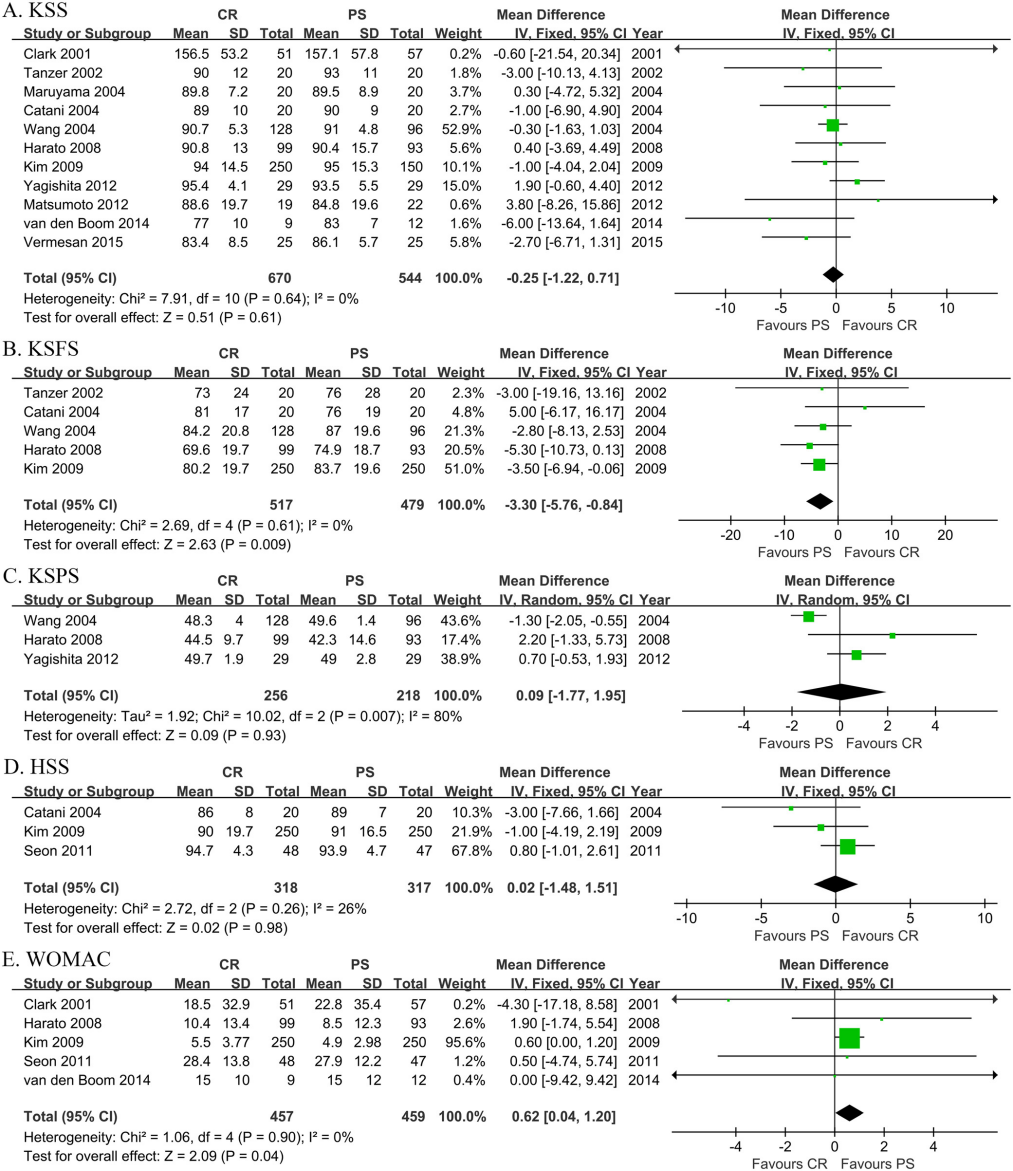
Meta-analysis of the clinical scores (Panel A-E). A. Meta-analysis of Knee Society knee Score (KSS). B. Meta-analysis of Knee Society function score (KSFS). C. Meta-analysis of Knee Society pain score (KSPS). D. Meta-analysis of Hospital for Special Surgery score (HSS). E. Meta-analysis of Western Ontario and McMaster Universities score (WOMAC). CR, Posterior Cruciate-retaining prostheses. PS, Posterior-Stabilized prostheses. Fixed, Fixed Effect model. Random, Random Effect model. SD, Standard Deviation. CI, Confidence Interval.

Meta-analysis of the clinical scores, including KSS, KSFS and KSPS, WOMAC score as well as HSS scores was shown in Fig 4. MD, CR minus PS was used to compare the relative effects. Only meta-analysis of the KSPS showed significant heterogeneity (Chi^2^ = 12.94, I^2^ = 77%, *p* = 0.005) and RE model was employed. There were no significant differences between CR and PS TKA among the KSS (FE, MD = -0.13, 95% CI -1.08 to 0.82, *p* = 0.79) and KSPS (RE, MD = 0.50, 95% CI -1.39 to 2.39, *p* = 0.60), as well as HSS score (FE, MD = 0.02, 95% CI -1.48 to 1.51, *p* = 0.98). However PS is superior to CR according to meta-analysis of KSFS score (FE, MD = -3.30.19, 95% CI -5.76 to -0.84, *p* = 0.009) and the WOMAC score (FE, MD = 0.62, 95% CI 0.04 to 1.20, *p* = 0.04).

### 3.4 Meta-analysis of clinical function

**Fig 5:**
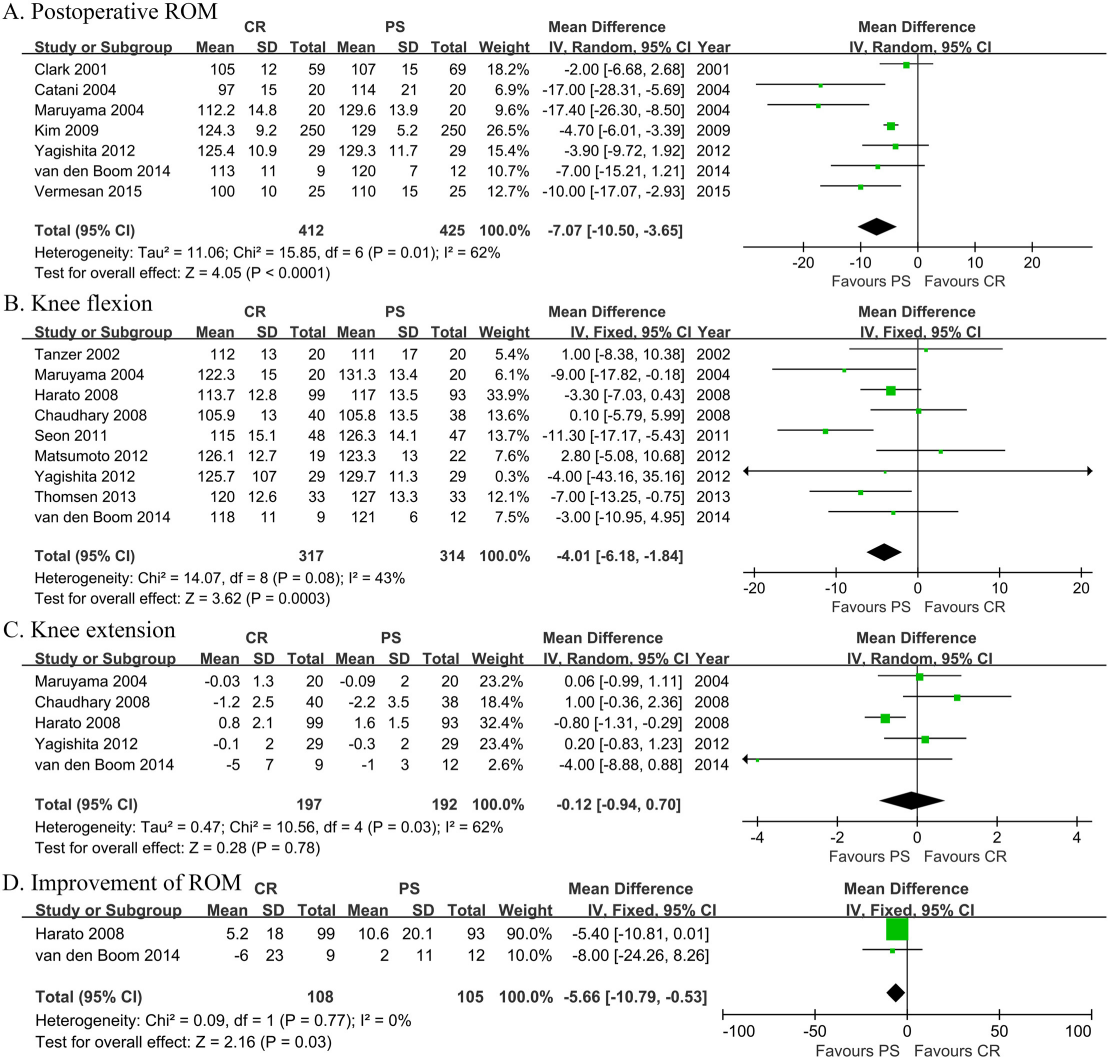
Meta-analysis of clinical function (Panel A-D). A. Meta-analysis of post-operative knee Range of Motion (ROM). B. Meta-analysis of knee flexion. C. Meta-analysis of knee extension. D. Meta-analysis of improvement of ROM.

Meta-analysis of clinical function, including postoperative ROM, knee flexion and extension was shown in Fig 5. There was significant heterogeneity among the ROM (Chi^2^ = 15.85, I^2^ = 62%, *p* = 0.01) and extension (Chi^2^ = 10.56, I^2^ = 62%, *p* = 0.03) analysis, for which RE model was employed. ROM (RE, MD = -7.07, 95% CI -10.50 to -3.65, *p*<0.0001) and flexion (FE, MD = -3.95, 95% CI -6.05 to -1.84, *p* = 0.0002) analyses indicated better function with PS versus CR. Knee extension analysis (RE, MD = -0.12, 95% CI -0.94 to 0.70, *p* = 0.78) showed no significant difference between the two groups. In addition, there were two studies (Fig 5D) that compared the change of ROM postoperatively, which showed better improvement of ROM with PS (FE, MD = -5.66, 95% CI -10.79 to -0.53, *p* = 0.03).

### 3.5 Meta-analysis of kinematic characteristics

**Fig 6:**
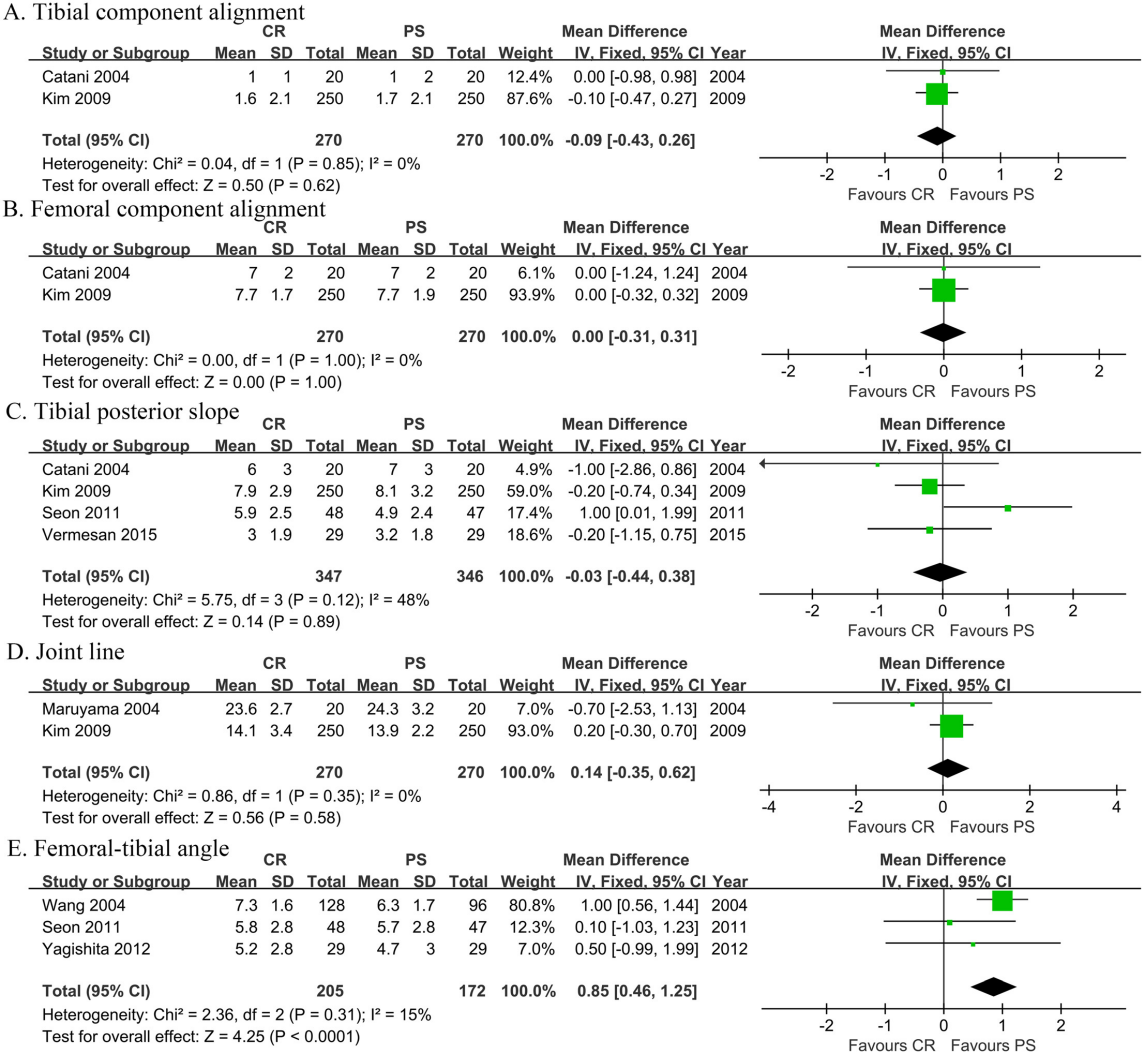
Meta-analysis of kinematic characteristics (Panel A-E). A. Meta-analysis of tibial component alignment. B. Meta-analysis of femoral component alignment. C. Meta-analysis of tibial posterior slope. D. Meta-analysis of joint line. E. Meta-analysis of femoral-tibial angle.

Meta-analysis of kinematic characteristics, including postoperative tibial and femoral component alignment, tibial posterior slope, joint line and femoral-tibial angle, was shown in Fig 6. No significant heterogeneity among the few studies focused on kinematics. With these less convincing meta-analyses, there were no significant difference pertaining to tibial component alignment (FE, MD = -0.09, 95% CI -0.43 to 0.26, *p* = 0.62), femoral component alignment (FE, MD = -0.00, 95% CI -0.31 to 0.31, *p* = 1.00), tibial posterior slope (FE, MD = -0.03, 95% CI -0.44 to 0.38, *p* = 0.89) and joint line (FE, MD = 0.14, 95% CI -0.35 to 0.62, *p* = 0.58). However, meta-analysis of postoperative femoral-tibial angle showed better alignment of femoral and tibial component in the PS group (FE, MD = 0.85, 95% CI 0.46 to 1.25, *p*<0.0001).

### 3.6 Meta-analysis of complications

**Fig 7:**
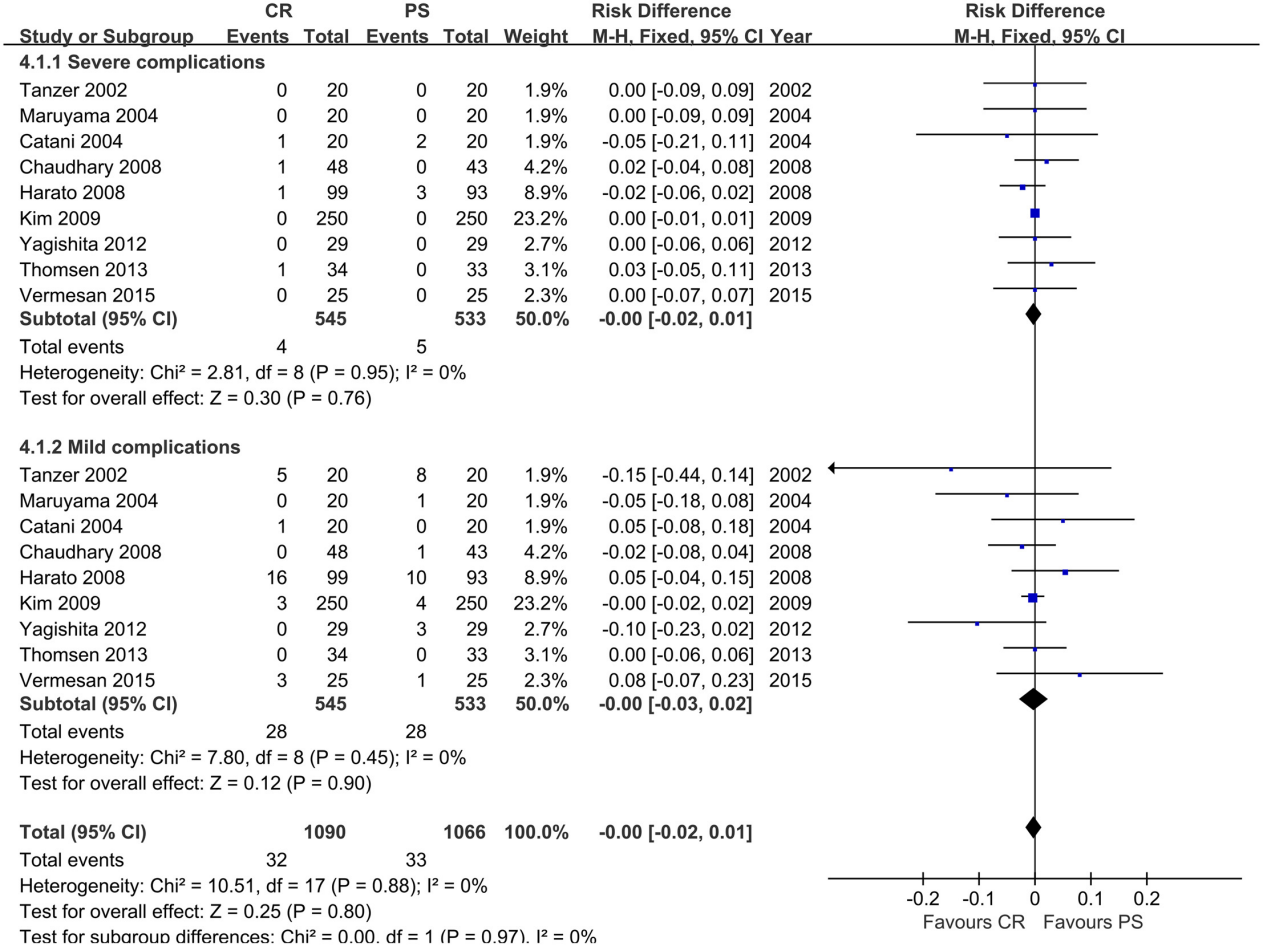
Meta-analysis of complications.

Subgroup analysis of complications was implemented with totally 545 patients in CR group and 533 patients in PS group (Fig 7). There is no significant differences incidence of both severe and mild complications between the two groups (FE, MD = 0.00, 95% CI -0.02 to 0.01, *p* = 0.80). The overall incidence of complication is about 6% and about 1% will need revision surgery or removal of the prosthesis for both groups.

## Discussion

In this study, we included totally 14 studies, with 2 more studies [12,13] than the former meta-analyses on this topic [22]. Besides, all studies included here were RCTs, which were known to provide the least biased evidence. Our study showed that although there were no significant differences in KSS, KSPS, HSS, post-operative extension angle, knee kinematic characteristics and rate of complication, PS TKA not only provided a better total ROM, improvement of ROM and flexion angle after TKA, but also had slightly higher scores in WOMAC and KSFS.

KSS, HSS and WOMAC are wildly used to evaluate the effectiveness of TKA in clinical practice. The data of all those included studies showed no differences between CR and PS TKA in post-operative KSS and HSS. WOMAC is slightly higher in PS group. However, the difference is so small that we think it is of no significance in clinical practice.

After TKA treatment, ROM of the operated knee is also a very important index when evaluating the effect. Most of the previous reports and meta-analyses found that post-operative ROM was better in PS TKA [8,9,10]. It was the same in our study. Included studies showed that PS TKA had a better ROM 6 months postoperatively. So did the postoperative knee flexion. But there was no significant difference in post-operative knee extension. Besides, we also analyzed the pre- to post-operative ROM improvement in CR and PS TKA, which was also better in PS TKA. Results from our analysis suggest that there is a difference in the mean ROM and flexion between CR and PS TKA, which favors PS TKA. This may be related to the excision of the posterior cruciate ligament and improved soft-tissue balancing. However, the clinical implication of this difference remains unclear, since the difference less than 5°-10°is thought to have no clinical impact [5, 19].

The femoral-tibial angle, position of the components, tibial posterior slope and level of the joint line were also compared in the current study. However, there is limited data can be extracted from previous RCTs. Based on the limited data, we found that CR and PS TKA have a similar outcome in knee kinematics. We think that post-operative knee kinematics may be affected by surgical technique more than the prosthesis design. Yet more studies on kinematic characteristics of CR and PS TKA need to be conducted

In the current study, we also compared the surgical complication rate in CR and PS TKA. We found that the two groups had no significant differences not only in mild complications that need no revision surgery such as DVT, superficial infection, but also in severe complications that need revision, which is similar to the previous study [8]. However, all the RCTs so far have a relatively short period of follow-up, and long-term follow-up studies of these two types of prostheses are necessitated.

Our study has several strengths. First of all, we searched all the three main medical databases, and only RCTs were included, studies such as retrospective control studies and RCTs [23, 24,25] that use a same prosthesis with or without PCL retained were excluded, while they were included in previous study [7,22]. Besides, all the databases were searched up to July 15, 2015, which covered the latest related articles in the field. Last but not least, our study analyzed clinical scores, ROM, knee kinematics and complications, and all the outcomes were showed in one article, and this might bring an all-around comparison between CR and PS TKA. We divided the complications into mild and severe group, divided by whether revision surgery was needed or not. This may help us to have a deep and exact understanding about different complication rate in CR and PS TKA.

However, there are some limitations to our study. First, this study was limited to the articles published in English, which had selection bias in language, and might miss some related RCTs that published in non-English. Secondly, different RCTs focused on different study objects, so the data in different RCTs varied. Especially in the study of knee kinematics, the number of related RCTs was limited. Finally, we could see that it varied in the way of comparison. In some studies, comparison was done within simultaneous bilateral TKA, with one knee underwent CR TKA and the other PS TKA on the same patient. It might be difficult for someone who had undergone bilateral TKA to evaluated clinical function and pain of each knee separately.

## Conclusion

Based on all currently RCTs on this topic, our study found that there were no differences between CR and PS TKA regarding to post-operative clinical knee scores, knee kinematics and the rate of complication including the rate of revision. However, PS does have an advantage over CR TKA in respects of post-operative knee flexion and total knee ROM. Whether this superiority affects further knee function or not still need further study. Whether this superiority matters or not in clinical practice still needs further investigation and longer follow-up.

## Supporting Information

S1 File The details of literature search strategies and the corresponding results. (DOC)

S2 File The judgment strategy of article quality assessment. (PDF)

S3 File PRISMA checklist. (DOC)
