# Supplementary material for: Posterior Cruciate Ligament Retention versus Posterior Stabilization for Total Knee Arthroplasty: A Meta-Analysis
Source: PLoS One. 2016 Jan 29;11(1):e0147865. doi: 10.1371/journal.pone.0147865 (PMC4732820; doi:10.1371/journal.pone.0147865)
Supplement: S1 File — (DOC) [file pone.0147865.s001.doc]

S1：The details of literature search strategies and the corresponding results

1. PubMed queries:

| Search | Query | Items found |
| --- | --- | --- |
| [#43](http://www.ncbi.nlm.nih.gov/pubmed/advanced) | Search **#5 ADN #42** | [1129](http://www.ncbi.nlm.nih.gov/pubmed/?cmd=HistorySearch&querykey=43) |
| [#42](http://www.ncbi.nlm.nih.gov/pubmed/advanced) | Search **#6 OR #7 OR #8 OR #9 OR #10 OR #11 OR #12 OR #13 OR #14 OR #15 OR #16 OR #17 OR #18 OR #19 OR #20 OR #21 OR #22 OR #23 OR #24 OR #25 OR #26 OR #27 OR #28 OR #29 OR #30 OR #31 OR #32 OR #33 OR #34 OR #35 OR #36 OR #37 OR #38 OR #39 OR #40 OR #41** | [1239](http://www.ncbi.nlm.nih.gov/pubmed/?cmd=HistorySearch&querykey=42) |
| [#41](http://www.ncbi.nlm.nih.gov/pubmed/advanced) | Search **cruciate sacrificed[Title/Abstract]** | [3](http://www.ncbi.nlm.nih.gov/pubmed/?cmd=HistorySearch&querykey=41) |
| [#40](http://www.ncbi.nlm.nih.gov/pubmed/advanced) | Search **cruciate sacrificing[Title/Abstract]** | [51](http://www.ncbi.nlm.nih.gov/pubmed/?cmd=HistorySearch&querykey=40) |
| [#39](http://www.ncbi.nlm.nih.gov/pubmed/advanced) | Search **cruciate sacrifice[Title/Abstract]** | [4](http://www.ncbi.nlm.nih.gov/pubmed/?cmd=HistorySearch&querykey=39) |
| [#38](http://www.ncbi.nlm.nih.gov/pubmed/advanced) | Search **cruciate ligament sacrificed[Title/Abstract]** Schema: **all** | [0](http://www.ncbi.nlm.nih.gov/pubmed/?cmd=HistorySearch&querykey=38) |
| [#37](http://www.ncbi.nlm.nih.gov/pubmed/advanced) | Search **cruciate ligament sacrificed[Title/Abstract]** | [0](http://www.ncbi.nlm.nih.gov/pubmed/?cmd=HistorySearch&querykey=37) |
| [#36](http://www.ncbi.nlm.nih.gov/pubmed/advanced) | Search **cruciate ligament sacrificing[Title/Abstract]** | [23](http://www.ncbi.nlm.nih.gov/pubmed/?cmd=HistorySearch&querykey=36) |
| [#35](http://www.ncbi.nlm.nih.gov/pubmed/advanced) | Search **cruciate ligament sacrifice[Title/Abstract]** | [3](http://www.ncbi.nlm.nih.gov/pubmed/?cmd=HistorySearch&querykey=35) |
| [#34](http://www.ncbi.nlm.nih.gov/pubmed/advanced) | Search **posterior cruciate ligament sacrificed[Title/Abstract]** Schema: **all** | [0](http://www.ncbi.nlm.nih.gov/pubmed/?cmd=HistorySearch&querykey=34) |
| [#33](http://www.ncbi.nlm.nih.gov/pubmed/advanced) | Search **posterior cruciate ligament sacrificed[Title/Abstract]** | [0](http://www.ncbi.nlm.nih.gov/pubmed/?cmd=HistorySearch&querykey=33) |
| [#32](http://www.ncbi.nlm.nih.gov/pubmed/advanced) | Search **posterior cruciate ligament sacrificing[Title/Abstract]** | [18](http://www.ncbi.nlm.nih.gov/pubmed/?cmd=HistorySearch&querykey=32) |
| [#31](http://www.ncbi.nlm.nih.gov/pubmed/advanced) | Search **posterior cruciate ligament sacrifice[Title/Abstract]** | [3](http://www.ncbi.nlm.nih.gov/pubmed/?cmd=HistorySearch&querykey=31) |
| [#30](http://www.ncbi.nlm.nih.gov/pubmed/advanced) | Search **posterior stabilized[Title/Abstract]** | [636](http://www.ncbi.nlm.nih.gov/pubmed/?cmd=HistorySearch&querykey=30) |
| [#29](http://www.ncbi.nlm.nih.gov/pubmed/advanced) | Search **posterior stabilizing[Title/Abstract]** | [38](http://www.ncbi.nlm.nih.gov/pubmed/?cmd=HistorySearch&querykey=29) |
| [#28](http://www.ncbi.nlm.nih.gov/pubmed/advanced) | Search **posterior stable[Title/Abstract]** Schema: **all** | [0](http://www.ncbi.nlm.nih.gov/pubmed/?cmd=HistorySearch&querykey=28) |
| [#27](http://www.ncbi.nlm.nih.gov/pubmed/advanced) | Search **posterior stable[Title/Abstract]** | [0](http://www.ncbi.nlm.nih.gov/pubmed/?cmd=HistorySearch&querykey=27) |
| [#26](http://www.ncbi.nlm.nih.gov/pubmed/advanced) | Search **cruciate substituted[Title/Abstract]** Schema: **all** | [0](http://www.ncbi.nlm.nih.gov/pubmed/?cmd=HistorySearch&querykey=26) |
| [#25](http://www.ncbi.nlm.nih.gov/pubmed/advanced) | Search **cruciate substituted[Title/Abstract]** | [0](http://www.ncbi.nlm.nih.gov/pubmed/?cmd=HistorySearch&querykey=25) |
| [#24](http://www.ncbi.nlm.nih.gov/pubmed/advanced) | Search **cruciate substituting[Title/Abstract]** | [115](http://www.ncbi.nlm.nih.gov/pubmed/?cmd=HistorySearch&querykey=24) |
| [#23](http://www.ncbi.nlm.nih.gov/pubmed/advanced) | Search **cruciate substitution[Title/Abstract]** | [12](http://www.ncbi.nlm.nih.gov/pubmed/?cmd=HistorySearch&querykey=23) |
| [#22](http://www.ncbi.nlm.nih.gov/pubmed/advanced) | Search **cruciate ligament substituted[Title/Abstract]** Schema: **all** | [0](http://www.ncbi.nlm.nih.gov/pubmed/?cmd=HistorySearch&querykey=22) |
| [#21](http://www.ncbi.nlm.nih.gov/pubmed/advanced) | Search **cruciate ligament substituted[Title/Abstract]** | [0](http://www.ncbi.nlm.nih.gov/pubmed/?cmd=HistorySearch&querykey=21) |
| [#20](http://www.ncbi.nlm.nih.gov/pubmed/advanced) | Search **cruciate ligament substituting[Title/Abstract]** | [19](http://www.ncbi.nlm.nih.gov/pubmed/?cmd=HistorySearch&querykey=20) |
| [#19](http://www.ncbi.nlm.nih.gov/pubmed/advanced) | Search **cruciate ligament substitution[Title/Abstract]** | [12](http://www.ncbi.nlm.nih.gov/pubmed/?cmd=HistorySearch&querykey=19) |
| [#18](http://www.ncbi.nlm.nih.gov/pubmed/advanced) | Search **posterior cruciate ligament substituted[Title/Abstract]** Schema: **all** | [0](http://www.ncbi.nlm.nih.gov/pubmed/?cmd=HistorySearch&querykey=18) |
| [#17](http://www.ncbi.nlm.nih.gov/pubmed/advanced) | Search **posterior cruciate ligament substituted[Title/Abstract]** | [0](http://www.ncbi.nlm.nih.gov/pubmed/?cmd=HistorySearch&querykey=17) |
| [#16](http://www.ncbi.nlm.nih.gov/pubmed/advanced) | Search **posterior cruciate ligament substituting[Title/Abstract]** | [16](http://www.ncbi.nlm.nih.gov/pubmed/?cmd=HistorySearch&querykey=16) |
| [#15](http://www.ncbi.nlm.nih.gov/pubmed/advanced) | Search **posterior cruciate ligament substitution[Title/Abstract]** | [4](http://www.ncbi.nlm.nih.gov/pubmed/?cmd=HistorySearch&querykey=15) |
| [#14](http://www.ncbi.nlm.nih.gov/pubmed/advanced) | Search **cruciate retained[Title/Abstract]** | [6](http://www.ncbi.nlm.nih.gov/pubmed/?cmd=HistorySearch&querykey=14) |
| [#13](http://www.ncbi.nlm.nih.gov/pubmed/advanced) | Search **cruciate retaining[Title/Abstract]** | [515](http://www.ncbi.nlm.nih.gov/pubmed/?cmd=HistorySearch&querykey=13) |
| [#12](http://www.ncbi.nlm.nih.gov/pubmed/advanced) | Search **cruciate ligament retained[Title/Abstract]** Schema: **all** | [0](http://www.ncbi.nlm.nih.gov/pubmed/?cmd=HistorySearch&querykey=12) |
| [#11](http://www.ncbi.nlm.nih.gov/pubmed/advanced) | Search **cruciate ligament retained[Title/Abstract]** | [0](http://www.ncbi.nlm.nih.gov/pubmed/?cmd=HistorySearch&querykey=11) |
| [#10](http://www.ncbi.nlm.nih.gov/pubmed/advanced) | Search **cruciate ligament retaining[Title/Abstract]** | [105](http://www.ncbi.nlm.nih.gov/pubmed/?cmd=HistorySearch&querykey=10) |
| [#9](http://www.ncbi.nlm.nih.gov/pubmed/advanced) | Search **posterior cruciate ligament retained[Title/Abstract]** | [4](http://www.ncbi.nlm.nih.gov/pubmed/?cmd=HistorySearch&querykey=9) |
| [#8](http://www.ncbi.nlm.nih.gov/pubmed/advanced) | Search **posterior cruciate ligament retaining[Title/Abstract]** | [103](http://www.ncbi.nlm.nih.gov/pubmed/?cmd=HistorySearch&querykey=8) |
| [#7](http://www.ncbi.nlm.nih.gov/pubmed/advanced) | Search **posterior cruciate ligament retaining[MeSH Terms]** Schema: **all** | [0](http://www.ncbi.nlm.nih.gov/pubmed/?cmd=HistorySearch&querykey=7) |
| [#6](http://www.ncbi.nlm.nih.gov/pubmed/advanced) | Search **posterior cruciate ligament retaining[MeSH Terms]** | [0](http://www.ncbi.nlm.nih.gov/pubmed/?cmd=HistorySearch&querykey=6) |
| [#5](http://www.ncbi.nlm.nih.gov/pubmed/advanced) | Search **#1 OR #2 OR #3 OR #4** | [19054](http://www.ncbi.nlm.nih.gov/pubmed/?cmd=HistorySearch&querykey=5) |
| [#4](http://www.ncbi.nlm.nih.gov/pubmed/advanced) | Search **total knee replacement[Title/Abstract]** | [3813](http://www.ncbi.nlm.nih.gov/pubmed/?cmd=HistorySearch&querykey=4) |
| [#3](http://www.ncbi.nlm.nih.gov/pubmed/advanced) | Search **total knee arthroplasty[Title/Abstract]** | [10440](http://www.ncbi.nlm.nih.gov/pubmed/?cmd=HistorySearch&querykey=3) |
| [#2](http://www.ncbi.nlm.nih.gov/pubmed/advanced) | Search **total knee arthroplasty[MeSH Terms]** | [14222](http://www.ncbi.nlm.nih.gov/pubmed/?cmd=HistorySearch&querykey=2) |
| [#1](http://www.ncbi.nlm.nih.gov/pubmed/advanced) | Search **total knee replacement[MeSH Terms]** | [14222](http://www.ncbi.nlm.nih.gov/pubmed/?cmd=HistorySearch&querykey=1) |

2. Embase queries:

| **No.** | **Query** | **Results** |
| --- | --- | --- |
| **#32** | **#3** AND **#31** | **883** |
| **#31** | **#4** OR **#5** OR **#6** OR **#7** OR **#8** OR **#9** OR **#10** OR **#11** OR **#12** OR **#13** OR **#14** OR **#15** OR **#16** OR **#17** OR **#18** OR **#19** OR **#20** OR **#21** OR **#22** OR **#23** OR **#24** OR **#25** OR **#26** OR **#27** OR **#28** OR **#29** OR **#30** | **1331** |
| **#30** | **'cruciate sacrificed'** | **3** |
| **#29** | **'cruciate sacrificing'** | **56** |
| **#28** | **'cruciate sacrifice'** | **6** |
| **#27** | **'cruciate ligament sacrifice'** | **4** |
| **#26** | **'cruciate ligament sacrificing'** | **26** |
| **#25** | **'cruciate ligament sacrificed'** | **3** |
| **#24** | **'posterior cruciate ligament sacrificed'** | **3** |
| **#23** | **'posterior cruciate ligament sacrificing'** | **19** |
| **#22** | **'posterior cruciate ligament sacrifice'** | **4** |
| **#21** | **'posterior stabilized'** | **695** |
| **#20** | **'posterior stabilizing'** | **40** |
| **#19** | **'posterior stable'** | **5** |
| **#18** | **'cruciate substituted'** | **0** |
| **#17** | **'cruciate substituting'** | **119** |
| **#16** | **'cruciate substitution'** | **12** |
| **#15** | **'cruciate ligament substitution'** | **17** |
| **#14** | **'cruciate ligament substituting'** | **22** |
| **#13** | **'cruciate ligament substituted'** | **0** |
| **#12** | **'posterior cruciate ligament substituted'** | **0** |
| **#11** | **'posterior cruciate ligament substituting'** | **19** |
| **#10** | **'posterior cruciate ligament substitution'** | **4** |
| **#9** | **'cruciate retaining'** | **525** |
| **#8** | **'cruciate retained'** | **7** |
| **#7** | **'cruciate ligament retained'** | **4** |
| **#6** | **'cruciate ligament retaining'** | **116** |
| **#5** | **'posterior cruciate ligament retained'** | **4** |
| **#4** | **'posterior cruciate ligament retaining'** | **114** |
| **#3** | **#1** OR **#2** | **11418** |
| **#2** | **'total knee arthroplasty'**/mj | **11418** |
| **#1** | **'total knee replacement'**/mj | **11418** |

3. Cochrane queries:

| ID | Search | Hits |
| --- | --- | --- |
| #1 | "total knee replacement":ti,ab,kw | 1317 |
| #2 | "total knee arthroplasty":ti,ab,kw | 1781 |
| #3 | #1 or #2 | 2487 |
| #4 | "posterior cruciate ligament retaining":ti,ab,kw | 18 |
| #5 | "posterior cruciate ligament retained":ti,ab,kw | 2 |
| #6 | "cruciate ligament retained":ti,ab,kw | 2 |
| #7 | "cruciate ligament retaining":ti,ab,kw | 18 |
| #8 | "cruciate retained":ti,ab,kw | 1 |
| #9 | "cruciate retaining":ti,ab,kw | 68 |
| #10 | "posterior cruciate ligament substitution":ti,ab,kw | 0 |
| #11 | "posterior cruciate ligament substituting":ti,ab,kw | 3 |
| #12 | "posterior cruciate ligament substituted":ti,ab,kw | 0 |
| #13 | "cruciate ligament substituting":ti,ab,kw | 4 |
| #14 | "cruciate ligament substitution":ti,ab,kw | 1 |
| #15 | "cruciate ligament substituted":ti,ab,kw | 0 |
| #16 | "cruciate substituting":ti,ab,kw | 27 |
| #17 | "cruciate substitution":ti,ab,kw | 1 |
| #18 | "cruciate substituted":ti,ab,kw | 0 |
| #19 | "posterior stable":ti,ab,kw | 0 |
| #20 | "posterior stabilizing":ti,ab,kw | 3 |
| #21 | "posterior stabilized":ti,ab,kw | 77 |
| #22 | "posterior cruciate ligament sacrifice":ti,ab,kw | 1 |
| #23 | "posterior cruciate ligament sacrificing":ti,ab,kw | 3 |
| #24 | "posterior cruciate ligament sacrificed":ti,ab,kw | 0 |
| #25 | "cruciate ligament sacrifice":ti,ab,kw | 1 |
| #26 | "cruciate ligament sacrificing":ti,ab,kw | 3 |
| #27 | "cruciate ligament sacrificed":ti,ab,kw | 0 |
| #28 | "cruciate sacrifice":ti,ab,kw | 1 |
| #29 | "cruciate sacrificing":ti,ab,kw | 3 |
| #30 | "cruciate sacrificed":ti,ab,kw | 0 |
| #31 | #4 or #5 or #6 or #7 or #8 or #9 or #10 or #11 or #12 or #13 or #14 or #15 or #16 or #17 or #18 or #19 or #20 or #21 or #22 or #23 or #24 or #25 or #26 or #27 or #28 or #29 or #30 | 155 |
| #32 | #3 and #31 | 141 |
