# Supplementary material for: Posterior Cruciate Ligament Retention versus Posterior Stabilization for Total Knee Arthroplasty: A Meta-Analysis
Source: PLoS One. 2016 Jan 29;11(1):e0147865. doi: 10.1371/journal.pone.0147865 (PMC4732820; doi:10.1371/journal.pone.0147865)
Supplement: S2 File — (PDF) [file pone.0147865.s002.pdf]

Characteristics of studies

Characteristics of included studies

Catani 2004

**Methods**	
**Participants**	
**Interventions**	
**Outcomes**	
**Notes**	

Risk of bias table

Bias	Authors' judgement	Support for judgement
Random sequence generation (selection bias)	Low risk	Forty patients with osteoarthritis of the knee received a unilateral arthroplasty using a cemented total knee prosthesis
Allocation concealment (selection bias)	Unclear risk	detailed information not mentioned
Blinding of participants and personnel (performance bias)	Low risk	detailed information not mentioned
Blinding of outcome assessment (detection bias)	Low risk	detailed information not mentioned
Incomplete outcome data (attrition bias)	Low risk	no patients lost follow-up
Selective reporting (reporting bias)	Low risk	The study protocol is available and outcomes have been reported in the pre-specified way
Other bias	Unclear risk	

Chaudhary 2008

**Methods**	
**Participants**	
**Interventions**	
**Outcomes**	
**Notes**	

Risk of bias table

Bias	Authors' judgement	Support for judgement
Random sequence generation (selection bias)	Low risk	The randomization codes, computer- generated in blocks of twenty subjects
Allocation concealment (selection bias)	Low risk	The randomization codes were stored in sequentially numbered opaque envelopes.
Blinding of participants and personnel (performance bias)	Low risk	Subjects gave informed consent for participation in the study prior to the randomization, and they were not informed about the group allocation.
Blinding of outcome assessment (detection bias)	Low risk	The range of motion of the knee was reassessed, independent of the orthopaedic surgeons
Incomplete outcome data (attrition bias)	Low risk	Of the 100 subjects, ninety-eight were alive at the time of the final assessment
Selective reporting (reporting bias)	Low risk	The range of motion of the knee was reassessed, independent of the orthopaedic surgeons
Other bias	Unclear risk	

Clark 2001

**Methods**	
**Participants**	
**Interventions**	
**Outcomes**	
**Notes**	

Risk of bias table

Bias	Authors' judgement	Support for judgement
Random sequence generation (selection bias)	Unclear risk	The randomization process was sub- jected to stratification by the surgeon at each cen- ter ensuring that each surgeon implanted approx- imately equal numbers of each type of prosthetic design.
Allocation concealment (selection bias)	Unclear risk	not mention
Blinding of participants and personnel (performance bias)	Unclear risk	not mention
Blinding of outcome assessment (detection bias)	Unclear risk	not mention
Incomplete outcome data (attrition bias)	Low risk	no patients loss follow-up

Selective reporting (reporting bias)	Low risk	The study protocol is available and outcomes have been reported in the pre-specified way.
Other bias	Unclear risk	

Harato 2008

**Methods**	
**Participants**	
**Interventions**	
**Outcomes**	
**Notes**	

Risk of bias table

Bias	Authors' judgement	Support for judgement
Random sequence generation (selection bias)	Low risk	Patients with degenerative osteoarthritis were selected as potential candidates for the study
Allocation concealment (selection bias)	Low risk	All knees were sequentially assigned to either the CR or PS treatment group, subsequent to enrollment into the study, using a closed en- velope randomization technique.
Blinding of participants and personnel (performance bias)	Low risk	Separate randomiza- tion schedules were prepared and executed at each individual surgical center. The randomization number for each patient was recorded on the Preoperative Medical Profile.
Blinding of outcome assessment (detection bias)	Unclear risk	not mention
Incomplete outcome data (attrition bias)	Low risk	30/222 loss follow-up or discontinued,which is low
Selective reporting (reporting bias)	Low risk	The study protocol is available and outcomes have been reported in the pre-specified way.
Other bias	Unclear risk	

Kim 2009

**Methods**	
**Participants**	
**Interventions**	
**Outcomes**	
**Notes**	

Risk of bias table

Bias	Authors' judgement	Support for judgement
Random sequence generation (selection bias)	Low risk	We enrolled 265 patients (530 knees) who had bilateral osteoarthritis that we considered to be severe enough for si- multaneous bilateral sequential total knee arthroplasty.
Allocation concealment (selection bias)	Low risk	Randomization to treatment with the NexGen CR-Flex or NexGen LPS-Flex total knee arthroplasty was accomplished with use of a sealed study number envelope, which was opened in the operating room before the skin incision was made.
Blinding of participants and personnel (performance bias)	Low risk	with use of a sealed study number envelope, which was opened in the operating room before the skin incision was made.
Blinding of outcome assessment (detection bias)	Low risk	All clinical data at the time of each follow-up were recorded and compiled by a clinical fellow (Y.W.C.) who was not part of the operative team and was blinded to allocation.
Incomplete outcome data (attrition bias)	Low risk	250 patients out of 256 finished the follow-up
Selective reporting (reporting bias)	Low risk	results were reported according to study design
Other bias	Unclear risk	

Maruyama 2004

**Methods**	
**Participants**	
**Interventions**	
**Outcomes**	
**Notes**	

Risk of bias table

Bias	Authors' judgement	Support for judgement
Random sequence generation (selection bias)	Low risk	The knees scheduled for TKAs were examined preoper- atively with varus-valgus stress radiographs, and those with significant fixed deformity were ex- cluded from the study.
Allocation concealment (selection bias)	Unclear risk	TKAs was randomly alternated among the 20 sub- jects.detailed steps were not mentioned

Blinding of participants and personnel (performance bias)	Unclear risk	not mentioned
Blinding of outcome assessment (detection bias)	Unclear risk	not mentioned
Incomplete outcome data (attrition bias)	Low risk	no patients were lost to follow-up.
Selective reporting (reporting bias)	Low risk	The study protocol is available and outcomes have been reported in the pre-specified way.
Other bias	Unclear risk	

Matsumoto 2012

**Methods**	
**Participants**	
**Interventions**	
**Outcomes**	
**Notes**	

Risk of bias table

**Bias**	**Authors' judgement**	**Support for judgement**
Random sequence generation (selection bias)	Low risk	To make a fair assessment and minimize the influences of clinical variables, the exclusion criteria consisted of knees with valgus deformity; severe bony defect, which needs bone graft or augmentation; revision total knee arthroplasty; active knee joint infection; or the need for bilateral total knee arthroplasty.
Allocation concealment (selection bias)	Low risk	we prospectively randomized 25 patients (25 osteoarthritic knees) to receive a CR TKA (NexGen CR Flex; Zimmer, Inc, Warsaw, Ind), and the other 25 patients (25 osteoarthritic knees) to receive a PS TKA (NexGen LPS Flex; Zimmer, Inc) according to the envelope method between 2003 and 2005.
Blinding of participants and personnel (performance bias)	Low risk	we prospectively randomized 25 patients (25 osteoarthritic knees) to receive a CR TKA (NexGen CR Flex; Zimmer, Inc, Warsaw, Ind), and the other 25 patients (25 osteoarthritic knees) to receive a PS TKA (NexGen LPS Flex; Zimmer, Inc) according to the envelope method between 2003 and 2005.
Blinding of outcome assessment (detection bias)	Low risk	The measurements were performed independently by the blinded authors.
Incomplete outcome data (attrition bias)	Low risk	41/50 patients comprised the study group

Selective reporting (reporting bias)	Low risk	The study protocol is available and outcomes have been reported in the pre-specified way.
Other bias	Unclear risk	

Seon 2011

**Methods**	
**Participants**	
**Interventions**	
**Outcomes**	
**Notes**	

Risk of bias table

**Bias**	**Authors' judgement**	**Support for judgement**
Random sequence generation (selection bias)	Low risk	Patients with osteoarthritis and a minimum preoperative range of motion of C90° were included. Patients with a history of open knee surgery requiring the placement of metallic implants or with a history of revision total knee arthroplasty, and those with a diagnosis other than osteoarthritis were excluded. Previous contralateral knee replacement was not considered an exclusion criterion unless accompanied by severe pain or restricted mobility.
Allocation concealment (selection bias)	Low risk	A flow diagram of this selection/exclusion process, prepared according to the CONSORT Guidelines
Blinding of participants and personnel (performance bias)	Low risk	Patients were<br>allocated to the CR group or the PS group using sealed envelopes when they were prepared and draped for surgery.
Blinding of outcome assessment (detection bias)	High risk	All range of motion and clinical data obtained at final follow-up visits were evaluated and recorded by two independent evaluators who were part of the surgical team.
Incomplete outcome data (attrition bias)	Low risk	95/102 patients finished the follow-up
Selective reporting (reporting bias)	Low risk	The study protocol is available and outcomes have been reported in the pre-specified way
Other bias	Unclear risk	

Tanzer 2002

**Methods**	
**Participants**	
**Interventions**	
**Outcomes**	
**Notes**	

Risk of bias table

Bias	Authors' judgement	Support for judgement
Random sequence generation (selection bias)	Unclear risk	A total of 37 consecutive patients with 40 knees were prospectively randomized to receive either...<br>not mentioned
Allocation concealment (selection bias)	Unclear risk	not mentioned
Blinding of participants and personnel (performance bias)	High risk	Patients were blinded to which knee design they had received. BUT<br>All surgeries were done by the senior author (M.T.).<br>The author performed operations. It is impossible to blind him.
Blinding of outcome assessment (detection bias)	Unclear risk	not mentioned
Incomplete outcome data (attrition bias)	Low risk	no lossed to follow up
Selective reporting (reporting bias)	Unclear risk	cannot access the original protocol of the study.
Other bias	Unclear risk	

Thomsen 2013

**Methods**	
**Participants**	
**Interventions**	
**Outcomes**	
**Notes**	

Risk of bias table

Bias	Authors' judgement	Support for judgement

Random sequence generation (selection bias)	Low risk	Patients were included in the study following informed consent to bilateral one-stage oper- ation and acceptance of two different TKA' s. Patients were excluded from having a one-stage bilateral procedure if they had a history of or objective findings indicating cardiopulmonary disease (i.e. ASA-score ⩾ 3)
Allocation concealment (selection bias)	Low risk	The left knee was oper- ated on first and a randomized computer-generated list decided the prosthesis to be used in either knee.
Blinding of participants and personnel (performance bias)	Low risk	Patients were not aware of which knee received which prosthesis.
Blinding of outcome assessment (detection bias)	Low risk	All assessments were performed by one investigator blinded to which knee had which prosthesis.
Incomplete outcome data (attrition bias)	Low risk	33/36 patients finished the follow-up
Selective reporting (reporting bias)	Low risk	The study protocol is available and outcomes have been reported in the pre-specified way
Other bias	Unclear risk	

van den Boom 2014

**Methods**	
**Participants**	
**Interventions**	
**Outcomes**	
**Notes**	

Risk of bias table

**Bias**	**Authors' judgement**	**Support for judgement**
Random sequence generation (selection bias)	Low risk	Inclusion criteria were as follows: (1) being a patient at Martini Hospital (Groningen, The Netherlands) with pri- mary symptomatic osteoarthritis of the knee; (2) a nonfixed varus or valgus deformity of <10°; (3) age between 55 and 85 years; (4) body mass index (BMI) lower than 35 kg/m2; (5) meeting criteria of class 1 or 2 of the American Society of Anesthesiologists (ASA); and (6) having given informed consent. Exclusion criteria were as follows: (1) inflamma- tory arthritis; (2) previous unicondylar knee replacement; (3) tibial/femoral osteotomy; (4) cruciate ligament recon- struction; (5) flexion <90°; (6) flexion contracture/exten- sion deficit >10°; (7) varus/valgus malalignment >10°; or (8) any other lower extremity disease.

Allocation concealment (selection bias)	Low risk	From each group, 12 patients were randomly selected (by convenience sample) to undergo gait analysis both pre- and postoperatively
Blinding of participants and personnel (performance bias)	Unclear risk	not mentioned
Blinding of outcome assessment (detection bias)	Low risk	Passive motion of the knee (flexion, extension, ROM) and the Knee Society Score (KSS) were scored by an independent exam- iner, who was blinded to the type of procedure that had been performed
Incomplete outcome data (attrition bias)	Low risk	21/24 patients finished follow-up
Selective reporting (reporting bias)	Low risk	The study protocol is available and outcomes have been reported in the pre-specified way
Other bias	Unclear risk	

Vermesan 2015

**Methods**	
**Participants**	
**Interventions**	
**Outcomes**	
**Notes**	

Risk of bias table

**Bias**	**Authors' judgement**	**Support for judgement**
Random sequence generation (selection bias)	High risk	Both primary and inflammatory diseases were included. Patients with previous contralateral knee replacement were also deemed eligible.
Allocation concealment (selection bias)	Unclear risk	not mentioned
Blinding of participants and personnel (performance bias)	Unclear risk	not mentioned
Blinding of outcome assessment (detection bias)	Unclear risk	not mentioned
Incomplete outcome data (attrition bias)	Low risk	no patients lost follow-up
Selective reporting (reporting bias)	Low risk	The study protocol is available and outcomes have been reported in the pre-specified way
Other bias	Unclear risk	

Wang 2004

**Methods**	
**Participants**	
**Interventions**	
**Outcomes**	
**Notes**	

Risk of bias table

**Bias**	**Authors' judgement**	**Support for judgement**
Random sequence generation (selection bias)	Unclear risk	The patients were randomly divided by the hospital admission into two groups.<br>During the course of this study, there was an inventory shortage for CS prosthesis, and this resulted in a disproportion in the number of patients in both groups.
Allocation concealment (selection bias)	Unclear risk	not mentioned.
Blinding of participants and personnel (performance bias)	Unclear risk	not mentioned.
Blinding of outcome assessment (detection bias)	Unclear risk	not mentioned.
Incomplete outcome data (attrition bias)	High risk	Forty-two patients with 43 knees including 29 that underwent CR and 14 that underwent CS were excluded for reasons including three deaths unrelated to knee surgery, three knees with deep wound infections, one above the knee amputation due to diabetic gangrene, one patient with cerebral vascular accident (CVA), one patient with Parkinson disease, one patient (2 knees) with colon cancer and 32 patients were lost to follow up.<br>32 patients were lost to follow up with unknown reasons.
Selective reporting (reporting bias)	Unclear risk	cannot access the original protocol
Other bias	Unclear risk	

Yagishita 2012

**Methods**	
**Participants**	
**Interventions**	
**Outcomes**	

**Notes**	

Risk of bias table

Bias	Authors' judgement	Support for judgement
Random sequence generation (selection bias)	Low risk	Patients were included if deformities of bilateral osteoarthritic knees were similar and if the grade of the Kellgren-Lawrence grading scale was the same in both knees. Patients were excluded if any augmenta- tion procedure was necessary because of significant bone defects of the femur and/or the tibia and if patients had osteoarthritis of the hip or the ankle, or neurologic deficits causing pain and restricted walking
Allocation concealment (selection bias)	Low risk	The patients were prospectively randomized to receive the CR design in the right knee or the left knee and the PS design in the other knee.
Blinding of participants and personnel (performance bias)	Unclear risk	not mentioned
Blinding of outcome assessment (detection bias)	Unclear risk	not mentioned
Incomplete outcome data (attrition bias)	Low risk	29/29 finished the follow-up
Selective reporting (reporting bias)	Low risk	The study protocol is available and outcomes have been reported in the pre-specified way
Other bias	Unclear risk	

Footnotes
