# Supplementary material for: Posterior Cruciate Ligament Retention versus Posterior Stabilization for Total Knee Arthroplasty: A Meta-Analysis
Source: PLoS One. 2016 Jan 29;11(1):e0147865. doi: 10.1371/journal.pone.0147865 (PMC4732820; doi:10.1371/journal.pone.0147865)
Supplement: S3 File — (DOC) [file pone.0147865.s003.doc]

| **Section/topic** | **#** | **Checklist item** | **Reported on page #** |
| --- | --- | --- | --- |
| **TITLE** | | |  |
| Title | 1 | Posterior Cruciate Ligament Retention versus Posterior Stabilization for Total Knee Arthroplasty: a Meta-analysis | 1,2 |
| **ABSTRACT** | | |  |
| Structured summary | 2 | Introduction: Although being debated for many years, the superiority of posterior cruciate-retaining (CR) total knee arthroplasty (TKA) and posterior-stabilized (PS) TKA remains to be controversial. We compare the knee scores, post-operative knee range of motion (ROM), radiological outcomes about knee kinematic and complications between posterior cruciate-retaining (CR) total knee arthroplasty (TKA) and posterior-stabilized (PS) TKA.  Methods: Literatures published up to August 2015 were searched in PubMed, Embase and Cochrane databases, and meta-analysis was performed using the software, Review Manager (RevMan) version 5.3.  Results: Totally 14 random control trials (RCTs) on this topic were included for the analysis, which showed that PS and CR TKA had no significant difference in Knee Society knee Score (KSS), pain score (KSPS), Hospital for Special Surgery score (HSS), kinematic characteristics including postoperative component alignment, tibial posterior slope and joint line, and complication rate. However, PS TKA is superior to CR TKA with respects to post-operative knee range of motion (ROM) [Random Effect model (RE), Mean Difference (MD)= -7.07, 95% Confidential Interval (CI) -10.50 to -3.65, p<0.0001), improvement of ROM (Fixed Effect model, MD=-5.66, 95% CI -10.79 to -0.53, p=0.03) and femoral-tibial angle [Fixed Effect model (FE), MD=0.85, 95% CI 0.46 to 1.25, p<0.0001].  Conclusions: There are no clinically relevant differences between CR and PS TKA in terms of clinical, functional, radiological outcome, and complications, while PS TKA is superior to CR TKA in respects of ROM, while whether this superiority matters or not in clinical practice still needs further investigation and longer follow-up. | 2 |
| **INTRODUCTION** | | |  |
| Rationale | 3 | Although being debated for many years, the superiority of posterior cruciate-retaining (CR) total knee arthroplasty (TKA) and posterior-stabilized (PS) TKA remains to be controversial. With the posterior cruciate ligament (PCL) retained, CR TKA was thought to be better regarding to post-operative knee proprioception and kinesthesia. While others believed that PS TKA had better range of motion (ROM) [3], easier in ligament balance, and more reliable femoral rollback. A systematic review and meta-analysis from 2012 compared knee scores, ROM, radiographic kinematics and complication between these two TKA designs, indicating that CR and PS TKA had no differences in knee scores, radiological outcomes and complications. Although PS TKA had a better ROM, it made no clinical difference. | 3 |
| Objectives | 4 | Following updates were published in 2013 and 2014 reporting similar outcomes in knee score and function. These studies included related random control trials （RCTs） till August 2011. Since then, there were more RCTs published to compare CR and PS TKA in clinical knee scores, function and complications. Taking this into consideration, we think it is necessary to make an update on this topic. The outcome measures for data aggregation were knee scores, post-operative ROM, radiological outcomes about knee kinematics and complications. | 3 |
| **METHODS** | | |  |
| Protocol and registration | 5 | This meta-analysis was not registered. We followed the general protocol of conducting a meta-analysis. Literature search, screening, selection and data extraction were performed independently by different authors. Disagreements were discussed and consulted until a consensus was made. The details can be found in “Materials and Methods” of the manuscript. |  |
| Eligibility criteria | 6 | (1) RCTs with at least 6 months follow-up. (2) Participants underwent primary TKA, unilateral or bilateral. (3) The operations were performed with PCL Retaining versus Posterior Stabilized prosthesis. The studies that compare PCL retaining versus sacrificing TKA using the same prosthesis were excluded. (4) End points were about clinical knee scores, clinical function, kinematic characteristics and complications. | 3 |
| Information sources | 7 | Literatures published up to August 2015 were searched in PubMed, Embase and Cochrane databases. | 3 |
| Search | 8 | We used key words “Total Knee Arthroplasty”, “Posterior Cruciate Ligament Retention”, “Posterior Cruciate Ligament Retaining”, “Posterior Stabilization”, “Posterior Cruciate Ligament Sacrificing”, and their synonyms to retrieve all studies about this topic. We also reviewed references of related reviews so that no studies were missed.  Literature search strategies for different databases can be found in the supplementary content uploaded with the manuscript. | 3 and supporting information Table S1 |
| Study selection | 9 | Title and abstract review was conducted firstly to rule out the apparently unrelated articles. Then the articles would be examined through the text to determine whether they should be included for the meta-analysis or not according to the eligibility criteria. Reviews and former meta-analyses about this topic were also kept for reference review. All screening works were conducted independently by two authors. Disagreements were discussed and consulted with corresponding author until a consensus was made. | 4 |
| Data collection process | 10 | Each study included was reviewed thoroughly to extract as much data as we can. With incomplete data in the published articles (e.g. only mean and range for specific measurements), we attempted to contact the authors for original data so that we can include more patients in this analysis. | 4 |
| Data items | 11 | Clinical scores, including Knee Society knee Score (KSS), function score (KSFS) and pain score (KSPS), The Western Ontario and McMaster Universities score (WOMAC) and Hospital for Special Surgery score (HSS), clinical function, including ROM, postoperative knee extension and flexion, kinematic characteristics, including postoperative component alignment, tibial posterior slope and joint line, and complications were all in the scope of this meta-analysis. | 4 |
| Risk of bias in individual studies | 12 | Quality assessment of included studies was conduct with the Risk of bias table in RevMan version 5.3 for RCTs. | FIG 2, and supporting information S2 |
| Summary measures | 13 | Standard Mean Difference (SMD) is employed as the effect measure for change of pain and edema due to the different unit system among the studies. | 4 |
| Synthesis of results | 14 | The software, Review Manager, make it much easier to synthesize the results. For each measurement, 95% CI and p value were calculated. p <0.05 was considered statistically significant. | 4 |

Page 1 of 2

| **Section/topic** | **#** | **Checklist item** | **Reported on page #** |
| --- | --- | --- | --- |
| Risk of bias across studies | 15 | We assessed the publication bias by visual inspection of funnel plot (with RevMan 5.3) | FIG 3 |
| Additional analyses | 16 | Subgroup analyses were performed with RevMan 5.3. See FIG 7 and the corresponding explanation in the “Results” part of our manuscript. | FIG 7 |
| **RESULTS** | | |  |
| Study selection | 17 | Please see FIGURE. 1, the flow diagram. | FIG. 1 |
| Study characteristics | 18 | Please see TABLE 1. | 5-9, table 1 |
| Risk of bias within studies | 19 | Quality assessment of included studies was conduct with the Risk of bias table in RevMan version 5.3 for RCTs. | FIG 2, and supporting information S2 |
| Results of individual studies | 20 | TABLE 1 | 5-9 |
| Synthesis of results | 21 | PS and CR TKA had no significant difference in Knee Society knee Score (KSS), pain score (KSPS), Hospital for Special Surgery score (HSS), kinematic characteristics including postoperative component alignment, tibial posterior slope and joint line, and complication rate. However, PS TKA is superior to CR TKA with respects to post-operative knee range of motion (ROM) [Random Effect model (RE), Mean Difference (MD)= -7.07, 95% Confidential Interval (CI) -10.50 to -3.65, p<0.0001), improvement of ROM (Fixed Effect model, MD=-5.66, 95% CI -10.79 to -0.53, p=0.03) and femoral-tibial angle [Fixed Effect model (FE), MD=0.85, 95% CI 0.46 to 1.25, p<0.0001]. | 10-12, FIG 4-7 |
| Risk of bias across studies | 22 | We assessed the publication bias by visual inspection of funnel plot (with RevMan 5.3) | FIG 3 |
| Additional analysis | 23 | Subgroup analyses were performed with RevMan 5.3. See FIG 7 and the corresponding explanation in the “Results” part of our manuscript. | FIG 7 |
| **DISCUSSION** | | |  |
| Summary of evidence | 24 | Please see the “discussion” part of the manuscript. | 12-14 |
| Limitations | 25 | First, this study was limited to the articles published in English, which had selection bias in language, and might miss some related RCTs that published in non-English. Secondly, different RCTs focused on different study objects, so the data in different RCTs varied. Especially in the study of knee kinematics, the number of related RCTs was limited. Finally, we could see that it varied in the way of comparison. In some studies, comparison was done within simultaneous bilateral TKA, with one knee underwent CR TKA and the other PS TKA on the same patient. It might be difficult for someone who had undergone bilateral TKA to evaluated clinical function and pain of each knee separately. | 14 |
| Conclusions | 26 | Based on all currently RCTs on this topic, our study found that there were no differences between CR and PS TKA regarding to post-operative clinical knee scores, knee kinematics and the rate of complication including the rate of revision. However, PS does have an advantage over CR TKA in respects of post-operative knee flexion and total knee ROM. Whether this superiority affects further knee function or not still need further study. Whether this superiority matters or not in clinical practice still needs further investigation and longer follow-up. | 14 |
| **FUNDING** | | |  |
| Funding | 27 | The research was supported in by grants from the Beijing Municipal Natural  Science Foundation (D121100004212001). |  |

*From:*  Moher D, Liberati A, Tetzlaff J, Altman DG, The PRISMA Group (2009). Preferred Reporting Items for Systematic Reviews and Meta-Analyses: The PRISMA Statement. PLoS Med 6(6): e1000097. doi:10.1371/journal.pmed1000097

For more information, visit: **www.prisma-statement.org**.

Page 2 of 2
